# Plasmon coupling in vertical split-ring resonator metamolecules

**DOI:** 10.1038/srep09726

**Published:** 2015-06-05

**Authors:** Pin Chieh Wu, Wei-Lun Hsu, Wei Ting Chen, Yao-Wei Huang, Chun Yen Liao, Ai Qun Liu, Nikolay I. Zheludev, Greg Sun, Din Ping Tsai

**Affiliations:** 1 Department of Physics, National Taiwan University, Taipei 10617, Taiwan; 2 School of Electrical and Electronic Engineering, Nanyang Technological University, Singapore 639798, Singapore; 3 Optoelectronics Research Centre and Centre for Photonic Metamaterials, University of Southampton, Southampton SO17 1BJ, UK; 4 TPI and Centre for Disruptive Photonic Technologies, Nanyang Technological University, Singapore 637371, Singapore; 5 Department of Engineering, University of Massachusetts Boston, Boston, MA 02125, U.S.A.; 6 Research Center for Applied Sciences, Academia Sinica, Taipei 11529, Taiwan

## Abstract

The past decade has seen a number of interesting designs proposed and implemented to generate artificial magnetism at optical frequencies using plasmonic metamaterials, but owing to the planar configurations of typically fabricated metamolecules that make up the metamaterials, the magnetic response is mainly driven by the electric field of the incident electromagnetic wave. We recently fabricated vertical split-ring resonators (VSRRs) which behave as magnetic metamolecules sensitive to both incident electric and magnetic fields with stronger induced magnetic dipole moment upon excitation in comparison to planar SRRs. The fabrication technique enabled us to study the plasmon coupling between VSRRs that stand up side by side where the coupling strength can be precisely controlled by varying the gap in between. The resulting wide tuning range of these resonance modes offers the possibility of developing frequency selective functional devices such as sensors and filters based on plasmon coupling with high sensitivity.

Plasmonic metamaterials composed of artificial sub-wavelength structures typically involving metal have gained tremendous interest during the past decade because of their extraordinary optical properties and potential applications^1^,^2^,^3^,^4^,^5^. These properties and applications of the metamaterials are intrinsically connected to the localized surface plasmon (SP) resonances (LSPR) arising from the collective oscillations of free electrons which induce strong electromagnetic fields adjacent to the artificial sub-wavelength metallic elements (referred to here as metamolecules) in the metamaterials^6^,^7^. Properties of metamaterials can be readily tailored by engineering their constituent metamolecules composed of subwavelength metal structures^8^. For instance, a metamolecule constructed with a pair of closely spaced plasmonic elements exhibits rather different optical response than those made of isolated ones^9^. The plasmonic coupling of metamolecules has been explored to achieve a number of applications, such as the Fano resonance^10^,^11^, toroidal dipolar response^12^,^13^, Rabi splitting^14^,^15^ and biosensors^16^. While these promising applications of metamaterials continue to extend beyond the reach of any conventional media, one cannot help notice that most of them are driven by the electric field of an incident electromagnetic wave^17^. It is nevertheless desirable to expand the optical properties of these metamaterials to include their responses to magnetic field as well. Magnetic coupling through mutual inductive effects has been studied in in-plane coupled split-ring resonators (SRRs)^18^,^19^,^20^,^21^,^22^, but the dipoles were still excited by the incident electric field in experiment. The fact that a majority of previous studies have mainly focused on the plasmonic properties in the metamaterials that are mostly derived from the dipole response to the electric field of the incident wave acting upon these metamaterials is the direct consequence of significant technical challenges in the fabrication of metamaterials because they are far more easily constructed with planar sub-wavelength elements on substrates^23^,^24^,^25^, and their magnetic dipole moments driven only by the electric field of incident electromagnetic wave are always perpendicular to the magnetic field of a normal incident wave, resulting in a weak interaction with the magnetic field^26^,^27^,^28^. Attempts have been made to address this issue with the use of multilayer metamaterials but the fabrication techniques are still challenging^29^,^30^,^31^,^32^. While the oblique incidence also allows for the magnetic response to be observed to a certain degree^33^,^34^, such an effect can be further enhanced with the fabrication of vertical split-ring resonator (VSRR) structures in which the metamolecules stand up vertically, leading to their magnetic dipoles that can not only be excited by the electric field, but also by the magnetic field directly under normal incidence^35^,^36^,^37^.

In this work, using a recently developed high precision alignment technique^38^, we have fabricated VSRRs which allowed us to study how incident electromagnetic fields interact with these VSRRs and to reveal the plasmon coupling between closely spaced VSRRs in dimer structures (metamolecules). We first numerically compare the magnetic plasmon excitation between isolated SRRs that are in either planar or vertical configuration. We then fabricate and measure spectral transmittance of isolated VSRRs to identify their magnetic resonance. Taking advantage of the flexibility in arranging VSRRs that stand up side by side where the coupling strength of their magnetic dipoles can be tuned far more efficiently with their spacing, we have observed electric and magnetic plasmon coupling of two VSRRs of different dimensions resulting in a range of resonance shift. The vertical configuration enable more densely packed metamolecules for enhanced plasmonic properties.

## Results

**Figure 1:**
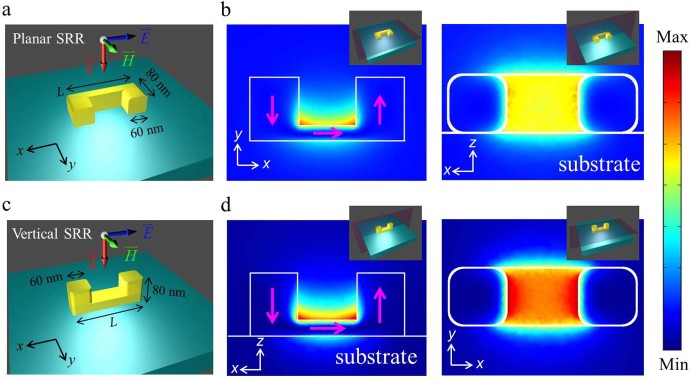
Field confinement and distribution in isolated SRR structures. Schematic diagrams of planar (a) and vertical SRR (c) of identical dimensions. Parameter *L* indicates the length of base rod (*L* = 195 nm). The induced magnetic energy density in arbitrary unit under normal incidence at plasmon resonance is shown in two perpendicular planes for comparison. Observation planes are shown as insets in (b) and (d). The side views (left) in (b) and (d) are for the plane that is centered along the SRR bas rods, the top views (right) in (b) and (d) are for the plane inserted between the base rod and the two prongs. Arrows in magenta indicate the direction of the induced surface current.

We have conducted numerical simulation using COMSOL to establish the comparison between single isolated planar and VSRR metamolecules of equal dimensions (base length *L* = 195 nm) as shown in Figs. 1a and 1c, respectively, under the excitation of a normal incident wave with its electric field polarized along the SRR gap (x-axis). In this configuration, planar SRRs are driven by the incident electric field only because the incident magnetic field is perpendicular to their magnetic dipoles which get induced only because of the bianisotropy^39^,^40^, the VSRRs, on the other hands, are excited by both electric and magnetic components of the incident wave and the effect of bianisotropy includes excitations of electric and magnetic dipoles by magnetic and electric fields, respectively. Considering gold SRRs placed on a glass (BK7) substrate, we have simulated the magnetic response of both SRRs. For planar SRRs, the magnetic response is induced by the oscillating electric current in the SRR due to its interaction with the incident electric field and a distribution of magnetic energy density is present within the SRR opening between the prongs (Fig. 1b). In comparison, the VSRR structure has a clear advantage in that it couples directly with not only the electric field but also the magnetic field under normal illumination. Our simulation result indicates that stronger magnetic energy density can indeed be obtained under the same dimensions and illumination condition (Fig. 1d).

**Figure 2:**
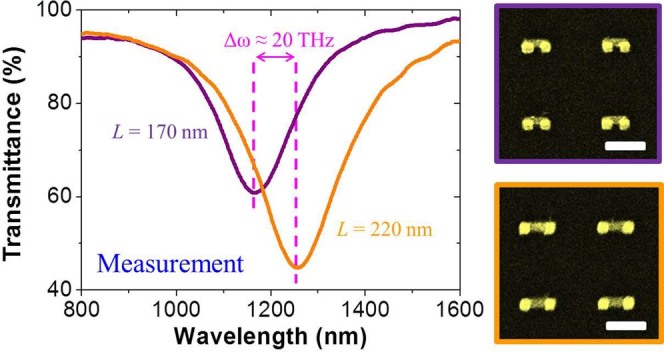
Electromagnetic response of isolated VSRR. Experimental transmittance spectra for isolated VSRR with 500 nm period in x and y directions on a glass (BK7) substrate. The purple and orange spectral lines correspond to the isolated VSRR with *L* = 170 nm and 220 nm, respectively. The resonance difference is about 20 THz in frequency indicated by the magenta dashed lines. The images on right represent the top view SEM pictures of the corresponding structures. Scale bars: 250 nm.

Inspired by the above simulation results, we proceed to fabricate VSRR structures with two different sizes as shown in Fig. 2 (right). The geometries for the two different structures are identical to the SRR dimensions used in simulation except the base length *L*. One sample has shorter base length of 170 nm while the other 220 nm. The reason for us to study these VSRRs of two different sizes is to establish the baseline for our next step in investigating the plasmon coupling between two closely spaced VSRRs of the same two sizes. The periodical lattice spacing in both samples has been chosen to be 500 nm to avoid coupling with its neighbors so that these VSRRs can be treated as being isolated. We have performed transmittance measurement on the two samples and the results are shown in Fig. 2 (left). There is a pronounced resonance dip for each isolated VSRR around 1200-nm wavelength which is the so-called LC resonance also referred to as the magnetic plasmon resonance because of the participation of magnetic dipole in the plasmon oscillation^41^. The resonance difference between the two isolated VSRRs of different dimensions is Δ*ω* ≈ 20 THz in the absence of plasmon coupling between them. The deeper transmittane dip observed from the lager VSRRs is the result of their greater area coverage density over the substrate relative to their smaller counterparts.

**Figure 3:**
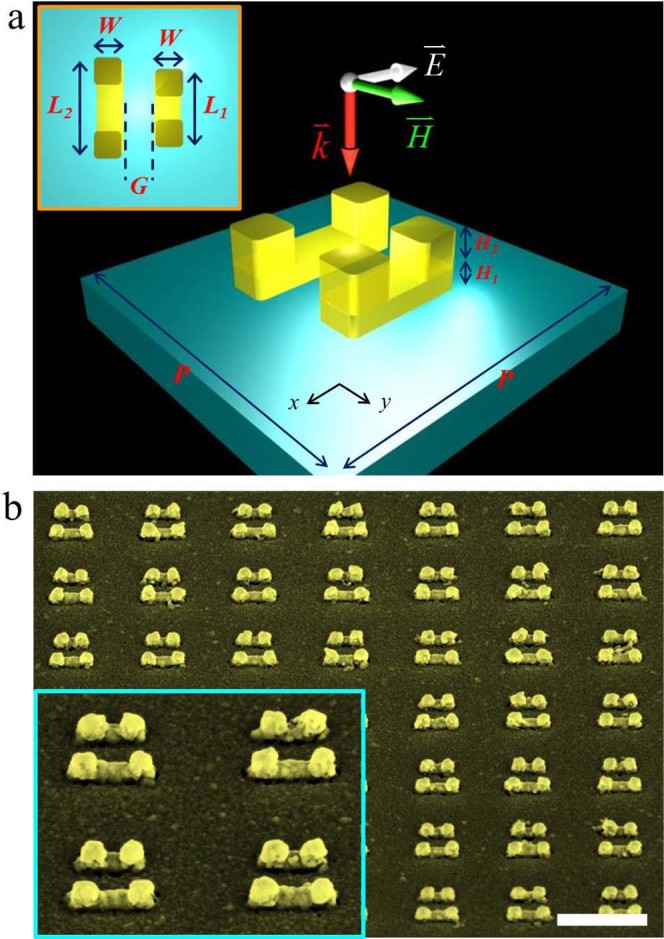
Geometry of VSRR dimer. (a) Schematic diagrams of VSRR dimer unit cell with designed parameters: *L_1_* = 170 nm, *L_2_* = 220 nm, *H_1_* = 20 nm, *H_2_* = 60 nm, *W* = 60 nm and *P* = 500 nm. A parameter *G* is introduced for the gap separation between VSRRs. (b) The 45° SEM micrograph with the zoom-in view (inset) for the sample with *G* = 50 nm. Scale bar: 500 nm.

We next investigate the resonance tuning of coupled VSRRs by fabricating a series of dimer samples with different spacing as shown schematically in Fig. 3a with the expectation to reveal the strong magnetic dipole coupling in vertical dimers because VSRRs can be placed much closer to each other. The two coupled VSRRs have the same geometries except different base lengths of *L_1_* = 170 nm and *L_2_* = 220 nm (the resonance position of each when isolated is shown in Fig. 2). As shown in Fig. 3a they are placed in parallel along x-axis with their centers aligned on y-axis. Figure 3b shows the SEM images (oblique views) of the gold VSRR dimer sample with 50-nm gap separation fabricated on a glass (BK7) substrate. The inset in Fig. 3b is an enlarged perspective view of four VSRR dimers with their two prongs sitting precisely on the two ends of the base rod.

**Figure 4:**
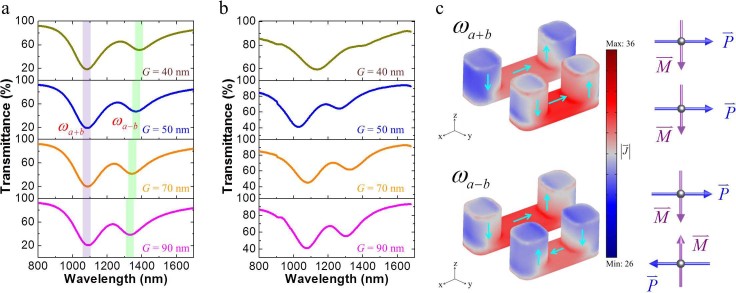
Electromagnetic spectra of VSRR dimers. (a) Simulation and (b) experimental transmittance spectra for VSRR dimers with various gaps. The colored shade highlights the position for each resonance mode. (c) Surface current density (in arbitrary unit and logarithmic scale) of the VSRR dimer with *G* = 50 nm under normal illumination at respective resonance wavelength *ω_a+b_* and *ω_a−b_* as indicated in (a). Arrows present the direction of induced surface current. Right: directions of induced electric and magnetic dipoles.

Four VSRR dimer samples with gap separations *G* of 40, 50, 70 and 90 nm are fabricated and measured. All samples have the same lattice constant of *P* = 500 nm in both x and y directions between each dimer unit cell (metamolecules) to avoid coupling between VSRRs from neighboring unit cell. Figure 4a represents the transmittance spectra simulated at four different gap separations between the two VSRRs where two transmittance dips emerge. The measurement (Fig. 4b) of these VSRR arrays reveals similar resonance features in reasonably good agreement with the simulation. The difference between the measurement and simulation is due to the VSRR size variation and roughness of fabricated samples that deviate from the exact dimensions and boundary condition of perfectly smooth structures used in the simulation. The two transmittance dips are clearly associated with the magnetic plasmon modes that originate from the two VSRRs of different dimensions. As the separation between the two VSRRs reduces, the coupling between them becomes stronger, shifting the two resonances further apart as revealed from the simulation and measurement in Figs. 4a and 4b, respectively.

## Discussion

**Figure 5:**
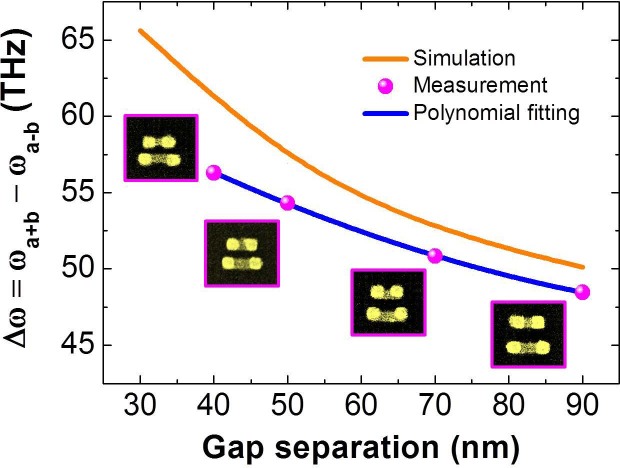
Bonding and anti-bonding splitting vs. the gap in VSRR dimer. Orange line and magenta dots correspond to simulation and measurement results, respectively. Blue line: a second-order polynomial fitting for experimental results. Insets represent the corresponding SEM image with gap sizes *G* = 40, 50, 70 and 90 nm between SRRs (left to right).

Plasmon hybridization theory^42^ has been proposed to reveal the origin of plasmon resonances of complex metal nanostructures as interactions between constituent elements much like the coupling between two closely spaced quantum structures where electron wavefunctions overlap. This theory has been proven successful in predicting and analyzing optical responses of assemblies of metal nanoparticles of various shapes including dimers among others^43^,^44^. The plasmon hybridization that has been reported so far primarily originates from interactions of electric and magnetic dipoles and higher-order multi-pole oscillations of individual nanoparticles that make up a complex nanostructure^10^,^45^. The VSRR dimer structures reported here offer a perfect venue to explore enhanced magnetic interaction between individual nanostructures that also influences the optical response of a composite metal structure. Indeed the VSRRs have much stronger magnetic coupling than those planar ones placed next to each other, and their coupling strength can be controlled by their spatial separation *G*. In the hybridization picture, each VSRR supports a dipole oscillation with its own plasmon resonance frequency at *ω_a_* or *ω_b_* depending on the VSRR dimensions, when two VSRRs are brought closer in the configuration shown in Fig. 3a their electric dipoles transversely couple to each other while the magnetic ones interact longitudinally, both contributing to the hybridization of resonance modes in the metamolecules that shifts the positions of original magnetic resonances *ω_a_* and *ω_b_* supported by the isolated VSRRs. It can be seen from the simulation result of the induced surface current distribution of the VSRR dimers that are separated by *G* = 50 nm under normal illumination in Fig. 4c that two resonance modes emerge from the coupled VSRRs, one associated with parallel induced electric currents in the two constituent VSRRs that enhances both electric and magnetic dipole moments, and the other with reduced moments from anti-parallel currents. The dominance of the electric coupling dictates that the “bonding” mode has out-of-phase electric dipoles, resulting in out-of-phase magnetic dipole moment oscillation as well (marked as *ω_a−b_* for their out-of-phase characteristic), while the “anti-bonding” mode (marked as *ω_a+b_*) has in-phase electric and magnetic dipoles. Since the two VSRRs have different dimensions, we have *ω_a_* ≠ *ω_b_*, and if we assume *ω_a_* < *ω_b_* the result of hybridization is to yield “bonding” and “anti-bonding” modes with their mode resonances separated further apart according to *ω_a−b_* < *ω_a_* < *ω_b_* < *ω_a+b_*. It is interesting to point out that the resonance at the longer wavelength (marked *ω*_a−b_) appears to be weaker as the gap separation becomes smaller, because this “bonding” mode originated from the two opposing electric and magnetic dipoles in the dimer as shown in Fig. 4c interacts weakly with the incident field. As a consequence, the resonance feature in the transmittance spectra at the shorter wavelength is always stronger than the one at the longer wavelength as shown in Figs. 4a and 4b. This plasmon hybridization of two unequal VSRRs shifts the plasmon resonances much like the coupling between two interacting semiconductor quantum dots (QDs) of different sizes. While each QD supports a confined state with a different energy, the result of coupling because of their electron wavefunction overlap is that the two confined states are pushed further apart. The amount of energy shifting reflects the coupling strength which depends on the geometries of the two QDs and is particularly sensitive to their spatial separation. We have also observed the similar behavior in the coupled VSRRs by systematically varying the separation *G* within a SRR dimer. Figure 5 shows the simulation and measurement of resonance frequency separation Δ*ω* = *ω_a+b_* − *ω_a−b_* of the “anti-bonding” and “bonding” for the four samples with VSRR spacing from 40 to 90 nm under normal illumination. The coupled resonance separation Δ*ω* is consistently greater than the resonance frequency difference (~20 THz) obtained from the transmittance measurement of the isolated VSRRs of same two different sizes shown in Fig. 2. As the spacing *G* between the VSRRs reduces, Δ*ω* increases rapidly with the decreasing *G*.

To summarize, we have fabricated a series of metamolecules consisting of either isolated VSRRs or their coupled dimers with different SRR spacing using e-beam lithography with high precision alignment technique. These VSRR metamolecules have the advantage of direct coupling to both the electric and magnetic components of the normal incident wave in comparison to their planar counterpart that only interacts with the electric field, resulting in stronger magnetic response. By conducting simulation and measurement of the optical transmittance, we have observed hybridization of magnetic plasmon modes associated with constituent VSRRs in metamolecules where bonding and anti-bonding modes emerged. We have found that the energy separation between the bonding and anti-bonding modes in metamolecules depends strongly on the gap separation in VSRR dimers. The tuning capability enabled by the magnetic plasmon mode coupling can be explored for developing frequency selective functional devices.

## Methods

### Fabrication of VSRRs

VSRR structures with different feature sizes are fabricated using electron beam lithography with high precision alignment technology. A 200 nm-thick 495 K PMMA (polymethyl methacrylate) layer was spin-coated at 4000 rpm on cover glass and then baked for 3 min at 180°C. The conductive polymer Espacer is then spin-coated at 1500 rpm over the PMMA layer to avoid the charging problem during the e-beam exposure process. An ELS-7000 electron beam lithography system (Elionix Inc., Tokyo, Japan) is used for exposure with 100 keV acceleration voltage and 30-pA current. The position of the VSRR base rod was defined on the PMMA resist relative to the two 100-nm-thick gold cross alignment marks which were first fabricated on the substrate for precise alignment during e-beam exposure process. After exposure, the sample was rinsed with de-ionized water to remove Espacer, then developed in solution of methyl isobutyl ketone (MIBK) and isopropyl alcohol (IPA) of MIBK:IPA = 1:3 for 60 seconds, rinsed again, this time with IPA, for 20 seconds, and blow-dried with nitrogen gas. Once the development of the resist was completed, a gold film with designed thickness was thermally deposited on the sample, and the un-patterned regions were removed using a lift-off process. Subsequently, the two VSRR prongs were fabricated in a similar fashion by the second e-beam exposure and lift-off process. The area of each fabricated structure is 75 × 75 μm^2^ on a cover glass substrate.

### Optical measurement and simulation

The spectra were measured by a self-assembled micro-spectrometer, and an inverted Olympus microscope IX-70 (10× IR objective with numerical aperture NA = 0.3, long working distance condenser with NA = 0.3, visible to near-infrared polarizer U68-750 from Edmoud Optics and 100 W halogen light source) equipped with two spectrometer (BTC111E for λ = 400 nm to λ = 1000 nm with ~0.5 nm resolution and BTC261E for λ = 900 nm to λ = 1700 nm with ~5 nm resolution) from B&W Tek, Inc. All transmittance spectra were normalized by an un-patterned region of the cover glass substrate.

### FEM Simulation

All simulation results were performed with the commercial software COMSOL Multiphysics by solving 3D Maxwell equations. Both isolated and coupled VSRR dimers are simulated with periodic boundary conditions under x-polarized light illumination. The refractive index of cover glass substrate is fixed at 1.51. The permittivity of gold in the near infrared regime is described by the Drude-Lorentz model with plasmon frequency *ω_p_* = 8.997 eV and damping constant *Γ_p_* = 0.14 eV, which is two times larger than that of the bulk value because of the surface scattering and grain effects.
